# Future directions in precognition research: more research can bridge the gap between skeptics and proponents

**DOI:** 10.3389/fpsyg.2014.00907

**Published:** 2014-08-22

**Authors:** Michael S. Franklin, Stephen L. Baumgart, Jonathan W. Schooler

**Affiliations:** ^1^Department of Psychological and Brain Sciences, University of California Santa BarbaraSanta Barbara, CA, USA; ^2^Theoretical and Applied Neurocausality LaboratorySanta Barbara, CA, USA

**Keywords:** precognition, retrocausality, psi, skepticism

## Introduction

Although claims of precognition have been prevalent across human history, it is no surprise that these assertions have been met with strong skepticism. Precognition, *the ability to obtain information about a future event, unknowable through inference alone, before the event actually occurs*, conflicts with the fundamental subjective experience of time asymmetrically flowing from past to future, brings into question the notion of free will, and contends with steadfast notions of cause and effect. Despite these reasons for skepticism, researchers have pursued this topic, and a large database of studies conducted under controlled laboratory conditions now exist. This work roughly spans from the 1930's (e.g., Rhine, 1938) up to this day (Bem, 2011; Mossbridge et al., 2014; Rabeyron, 2014). The accumulated evidence includes significant meta-analyses of forced-choice guessing experiments (Honorton and Ferrari, 1989), presentiment experiments (Mossbridge et al., 2012), and recent replications from Bem (2011, discussed below; Bem et al., 2014).

Perhaps most central to the recent debate regarding the existence of precognition is work by Bem (2011). Bem (2011) time-reversed several classic psychology effects (e.g., studying after instead of before a test; being primed after, instead of before responding) and found evidence across nine experiments supporting precognition. Given the sound methodology and publication at a high-impact mainstream psychology journal, *Journal of Personality and Social Psychology*, this work has prompted the attention of psychologists; and, not surprisingly, the response has been skeptical (Rouder and Morey, 2011; Wagenmakers et al., 2011). While we acknowledge skepticism and close scrutiny is vital in reaching consensus on this topic, given the equivocation surrounding the results, we propose that more research is needed. In particular, we suggest that applied research designs that allow for the prediction of meaningful events ahead of time can move this debate forward. Since it is not obvious how experiments that do not require explicit “guessing” of future events could be used for this goal, we give a general overview of two methodologies designed toward this aim.

## Physical implausibility

It is not unexpected that psychologists are most skeptical of precognition (Wagner and Monnet, 1979). This is likely due to their knowledge of the many illusions and biases that influence perception and memory. However, putting these cognitive biases aside, this work is often dismissed out of hand under the assumption that precognition would require overturning basic and essential physical and psychological tenets. Schwarzkopf (2014) illustrates this position:

“… the seismic nature of these claims cannot be overstated: future events influencing the past breaks the second law of thermodynamics… It also completely undermines over a century of experimental research based on the assumption that causes precede effects”

Some clarification is needed here. From a physics perspective, except for several processes studied in high-energy physics (such as B meson decay), non-thermal physics is time-symmetric, perhaps allowing the possibility of precognitive effects. The formalism of time symmetric physics has been used, for example, in the Wheeler-Feynman absorber theory of radiation (Wheeler and Feynman, 1945) as well as in the transactional interpretation of quantum mechanics (Cramer, 1986), in which quantum wavefunction collapse is described as being due to an interaction between advanced waves (traveling backwards-in-time) and retarded waves (traveling forwards-in-time). With regards to precognition, Bierman (2008) has proposed that coherent conditions present in the human brain allow the fundamental time symmetry of physics to manifest itself.

Some quantum mechanical experiments can be interpreted as showing retrocausal influence where a decision at a future time seems to affect a past time. One example is Wheeler's delayed-choice experiment in which the way a photon travels through an interferometer (wave-like or particle-like) appears to be affected by a measurement decision made at a later time (Wheeler, 1984; Jacques et al., 2007). However, information transfer into the past (retrocausal signaling), as opposed to influence without information transfer, remains controversial since it has not yet been demonstrated experimentally. That said, there is no physical law which precludes retrocausal information transfer. There has been some effort put into experimental realization of retrocausal signaling. Cramer proposed that standard quantum mechanics allows the construction of a retrocausal signaling machine using quantum optical interferometry (Cramer, 2007). Though Cramer's work has reached an impasse (Cramer, 2014), an approach of using entangled systems for retrocausal communication may reveal a physical explanation for precognition. Lastly, it is worth noting, that ultimately whether any given theory can accommodate precognition or not is irrelevant; what is relevant are the data.

## Reliability concerns

Although it appears premature to rule out precognition from a physics standpoint, there have been concerns regarding the reliability of precognitive effects. In essence, the question boils down to whether there are in fact small, yet real, precognitive effects that are hard to pin down and require further study to isolate, or, whether the evidence for precognition is based on false-positives emerging due to biases in the research process. For a recent overview of these issues in psychology see the November, 2012 issue of *Perspectives on Psychological Science*. Interestingly, a recent commentary (Jolij, 2014) notes the similarity between precognitive effects and those in social priming research. Indeed, both research areas report small effect sizes, replication difficulty, and specific “boundary” conditions (covariates) that moderate the effect (Wilson, 2013). Although researchers point toward meta-analyses to bolster their position, meta-analyses are also susceptible to bias and rarely lead to headway in controversial areas (Ferguson, 2014). The resemblance between precognitive effects and those seen in the mainstream psychological literature has been used to leverage support for precognition (e.g., Cardeña, 2014); however, the difficulties of replicating other paradigms in psychology seems a dubious source of solace for the challenge of replicating precognition findings. Moreover, even if precognition results were robustly replicated as some meta-analyses have suggested, there is always the concern that there is some artifact driving the effect. As such, we suggest new directions for future research in precognition; one that can simultaneously address concerns about the robustness of the effects and the possibility that they are driven by unrecognized artifacts.

## Future directions in precognition research

**Figure 1:**
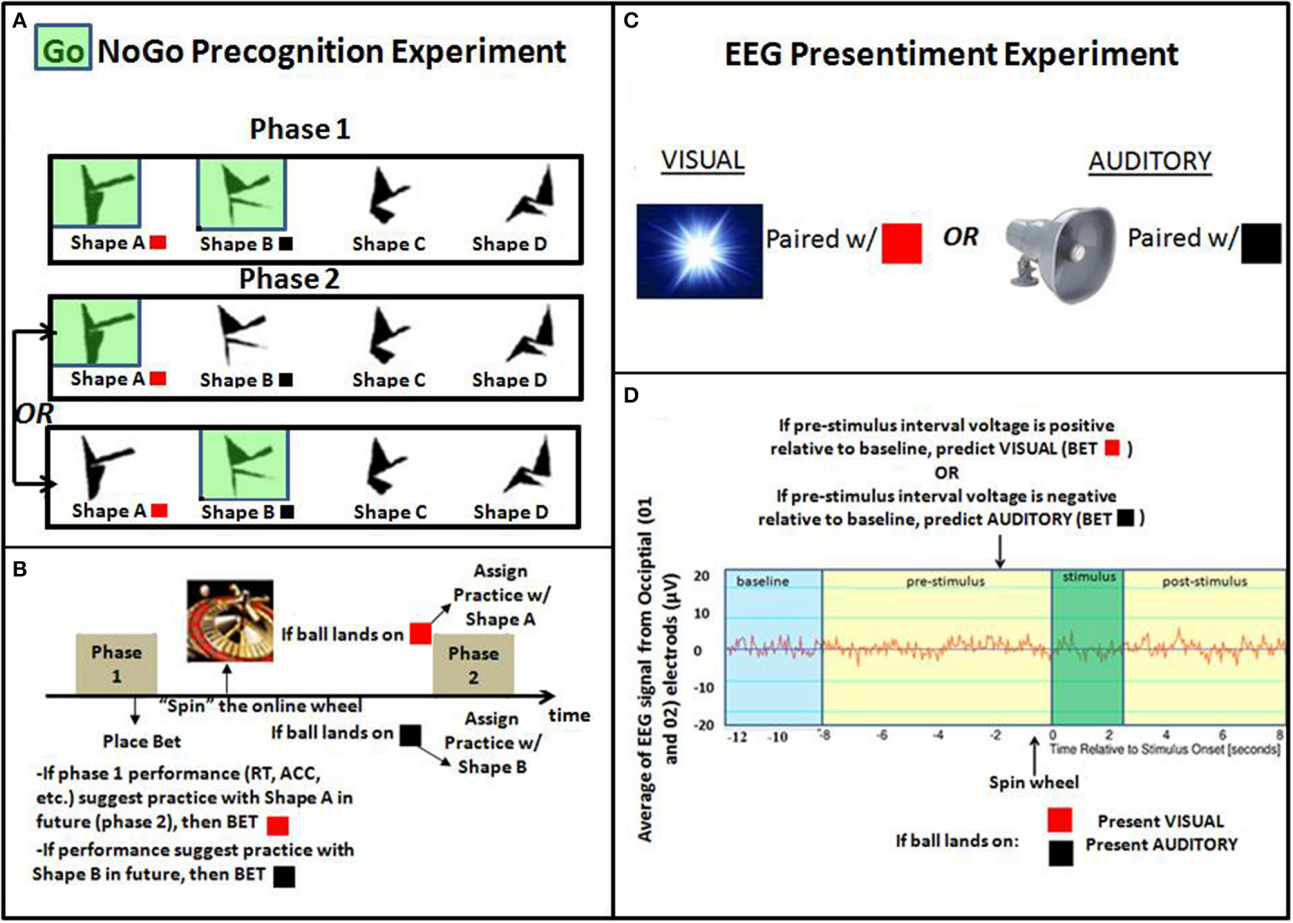
**The left side displays the experimental design of two-phase Go-NoGo precognition task: (A) 4 random polygons are displayed individually on screen for 1 s at a time**. Shape A is (arbitrarilly) associated with RED, and Shape B is associated with BLACK. During phase 1 all participants are told to press the spacebar only when shape A and B appear (the “Go” shapes, colored green), and withhold responses to shapes C and D while these responses and reaction times are recorded. In phase 2, particpants only respond to one “Go” shape. As seen in **(B)** the phase 2 shape is determined by a roulette spin outcome^2^. As such, the precognitive influence of phase 2 practice on phase 1 performance (e.g., improved detection of the shape practiced in the future) would allow for a real-time prediction of the future practice shape, and hence the future roulette spin outcome. On the right, is an overview of the experimental design of the “applied” EEG presentiment experiment: **(C)** Short duration visual or auditory stimuli are randomly presented to participants (equal probability). For the purposes of roulette spin prediction, each stimulus type is arbitrally associated with an outcome (Visual—RED, Auditory—BLACK) **(D)** EEG is continuously recorded from occipital electrodes (O1/O2). Prior to assigning a stimulus, a prediction is made based on a comparsion of the pre-stimlus interval to the baseline. Specfically, if voltage is positive relative to baseline, predict VISUAL (bet RED); if voltage is negative relative to baseline, predict AUDITORY (bet BLACK).

What would provide the most compelling evidence for skeptics? Ultimately, we realize that the most convincing demonstration would be to show tangible effects applied in real-world settings. If a paradigm can make accurate predictions about events that people consider important and are incapable of predicting using standard means, then the significance of the paradigm becomes self-evident. Perhaps most compelling would be if an experiment could be devised to predict games of chance and/or the whether it will be a good or bad day on the stock market. Although a few reports exist in the literature of precognitive applications, in particular those that utilize associative remote viewing (predicting silver future: Puthoff, 1984; stock market; Smith et al., 2014), there has not been a single replicable methdology that has translated into consistent winnings in games of chance. Below we give a brief overview of two experiments designed to predict the outcome of random^1^ binary events in real-time (specifically, the outcome of a roulette spin, black vs. red, excluding green; see Figure 1).

The left side of Figure 1 presents a general overview of one approach. This experiment is based on work designed to examine whether extended future practice in some domain can extend backwards in time to influence prior performance. The original experiment designed toward this aim used a novel 2-phase Go-NoGo experiment (Franklin, 2007). In phase 1 of the experiment, all participants complete an identical Go-NoGo task in which individual shapes are presented for a second, one at a time, on a computer screen. Each stimulus either requires a response (“Go”) or not (“NoGo”). Participants are told to respond (using the spacebar) to shapes A and B and withhold responses to shapes C and D. In phase 2, participants are randomly divided into 2 groups with each group responding exclusively to a single shape (A or B). The rationale is akin to the subtraction method/additive factors methodology (Sternberg, 1969). If phase 1 performance is influenced by only past experience, then there should be no difference in reaction times or accuracy based on future condition assignment. If, however, phase 1 performance is influenced not only by past experience, but future experience as well, systematic differences in performance based on phase 2 condition assignment should emerge. As seen in Figure 1B, by mapping shapes A and B to outcomes of the roulette spin (RED and BLACK), it should be possible (assuming a genuine precognitive effect) to use phase 1 performance to predict the roulette spin outcome before the wheel is spun.

Next we describe an experiment using EEG to detect predictive anticipatory activity (PAA; Mossbridge et al., 2014); also known as presentiment, the finding that various physiological measures of arousal are higher preceding the onset of emotionally charged vs. neutral pictures that are randomly presented (Bierman and Radin, 1997; Radin, 1997; Bierman and Scholte, 2002; Spottiswoode and May, 2003; Mossbridge et al., 2012). The specific methodology below extends work reported in Radin (2011), in which the pre-stimulus EEG activity of experienced meditators was found to differ significantly in response to light flashes and auditory tones. As seen in Figure 1, by mapping the light flash and auditory tone to a binary target (RED vs. BLACK roulette spin) and by evaluating baseline and pre-stimulus EEG potentials in real-time, it should be possible to predict the state of a future random target, allowing above-chance retrocausal communication. Similar to the first experiment design, the results of the prediction can be compared against chance (50%) with an exact binomial test. Currently, pilot testing with this basic design is underway, along with additional testing to assess whether a stimulus (flash vs. tone) triggered by the appropriate symmetric pre-stimulus response (a “neurofeedback” condition; e.g., flash delivered when occipital EEG increases) can condition response patterns in anticipation to random stimuli determined by roulette spin; allowing for a retrocausal Brain Computer Interface (BCI).

The design presented in Figure 1 has the benefit of more protection against anticipation/learning strategies (there is only one future event). Also, extended exposure to the future stimulus may strengthen the effect and allow for more time between the prediction, bet and outcome. Although the EEG experiment relies on fewer data points for each prediction, this method could lead to BCI applications and be more powerful due to the large number of trials collected within and across participants. Altogether, there appears to be no inherent confound in either design given sufficient sample size—i.e., we know of no conventional confound that could lead to consistent above chance prediction in real time of a roulette spin. As such, both designs are worth exploring in future research.

## Final thoughts

Despite the accumulated data, and recent positive findings in the literature, significant controversy remains regarding the interpretation of the evidence for the existence of precognition. Proponents find the combined results as compelling evidence in support of precognition, with similar (small) effect sizes to those reported throughout the psychological literature. Skeptics, however, question potential methodological and/or analytical confounds in those studies, as well as the physical plausibility of precognition. Both, however, agree regarding the profound implications if these bold claims are true. We suggest that although the current state of evidence does not quite merit proponents' strong claim of having demonstrated replicable precognition in the laboratory, the accumulated experimental evidence, combined with advances in theoretical physics, warrant further research. We believe the most effective way forward is through the development of paradigms that use software in real-time to predict meaningful future outcomes before they occur. As others have noted (Mossbridge et al., 2014) a new technology that uses behavior and/or physiology to consistently predict random future events above chance would certainly be a “game-changer.”

### Conflict of interest statement

The authors declare that the research was conducted in the absence of any commercial or financial relationships that could be construed as a potential conflict of interest.

## Footnotes

^1^Although there is an important distinction between truly random vs. pseudorandom selection, since any genuine precognitive effect of future stimuli on past behavior/physiology should be independent of selection method, we do not distinguish between these for the purposes of this overview.

^2^If the ball lands on green, re-spinning would occur until it lands on either black or red.
